# Transarterial Chemoembolization with Epirubicin-Loaded Microspheres for Hepatocellular Carcinoma: A Prospective, Single-Arm, Multicenter, Phase 2 Study (STOPPER Trial)

**DOI:** 10.1007/s00270-024-03666-4

**Published:** 2024-02-27

**Authors:** Hai-Dong Zhu, Xiao Li, Jun-Hui Sun, Xu Zhu, Zhao-Yu Liu, Hai-Liang Li, Jian Lu, Zhi-Ping Yan, Guo-Liang Shao, Xiao-Feng He, Min Chao, Li-Gong Lu, Bin-Yan Zhong, Rui Li, Qi Zhang, Gao-Jun Teng

**Affiliations:** 1 Center of Interventional Radiology and Vascular Surgery, Department of Radiology, Nurturing Center of Jiangsu Province for State Laboratory of AI Imaging & Interventional Radiology (Southeast University), Basic Medicine Research and Innovation Center of Ministry of Education, Zhongda Hospital, Medical School, Southeast University, 87 Dingjiaqiao Road, Nanjing, 210009 China; 2 Department of Interventional Therapy, National Cancer Center/National Clinical Research Center for Cancer/Cancer Hospital, Chinese Academy of Medical Sciences and Peking Union Medical College, Beijing, China; 3 Division of Hepatobiliary and Pancreatic Surgery, Hepatobiliary and Pancreatic Interventional Treatment Center, The First Affiliated Hospital, Zhejiang University School of Medicine, Hangzhou, China; 4 Interventional Therapy Department, Peking University Cancer Hospital and Institute, Beijing, China; 5 Department of Radiology, Shengjing Hospital of China Medical University, Shenyang, China; 6 Department of Minimal-Invasive Intervention, The Affiliated Cancer Hospital of Zhengzhou University, Zhengzhou, China; 7 Department of Interventional Radiology, Zhongshan Hospital, Fudan University Shanghai Institution of Medical Imaging, Fudan University, Shanghai, China; 8 Department of Intervention, The Cancer Hospital of the University of Chinese Academy of Sciences, Zhejiang Cancer Hospital, Institute of Basic Medicine and Cancer, Chinese Academy of Sciences, Hangzhou, China; 9 Division of Vascular and Interventional Radiology, Department of General Surgery, Nanfang Hospital, Southern Medical University, Guangzhou, China; 10 Department of Radiology, Second Affiliated Hospital of School of Medicine, Zhejiang University, Hangzhou, China; 11 Zhuhai Interventional Medical Center, Zhuhai Precision Medical Center, Zhuhai People’s Hospital, Zhuhai Hospital Affiliated with Jinan University, Jinan University, Zhuhai, China; 12 Department of Interventional Radiology, The First Affiliated Hospital of Soochow University, Soochow University, Suzhou, China

**Keywords:** Transarterial chemoembolization, Hepatocellular carcinoma, TANDEM microspheres, Epirubicin

## Abstract

**Purpose:**

While the role of drug-eluting beads transarterial chemoembolization (DEB-TACE) for hepatocellular carcinoma (HCC) is established, questions regarding appropriate bead size for use in patients remain. This trial evaluated the effectiveness and safety of DEB-TACE using small-size (≤ 100 μm) microspheres loaded with epirubicin.

**Materials and Methods:**

This prospective, single-arm, multicenter study enrolled patients diagnosed with HCC who underwent DEB-TACE using 40 (range, 30–50), 75 (range, 60–90), or 100 (range, 75–125) μm epirubicin-loaded microspheres (TANDEM microspheres, Varian Medical). Bead size was at the discretion of treating physicians and based on tumor size and/or vascular structure. The primary outcome measure was 6-month objective response rate (ORR). Secondary outcome measures were 30-day and 3-month ORR, time to tumor progression and extrahepatic spread, proportion of progression-free survival and overall survival (OS) at one year, and incidence of treatment-associated adverse events.

**Results:**

Data from 108 patients from ten centers was analyzed. Six-month ORR was 73.3 and 71.3% based on European association for the study of the liver (EASL) and modified response evaluation criteria in solid tumors (mRECIST) criteria, respectively. Thirty-day ORR was 79.6% for both EASL and mRECIST criteria with 3-month ORR being 80.0 and 81.0%, respectively, for each criteria. One-year PPF and OS rate were 60.3 and 94.3%. There was a total of 30 SAEs reported to be likely to definitely associated with microsphere (*n* = 9), epirubicin (*n* = 9), or procedure (*n* = 12) with none resulting in death.

**Conclusion:**

DEB-TACE using epirubicin-loaded small-sized (≤ 100 μm) microspheres demonstrates promising local tumor control and acceptable safety in patients with HCC.

**Trial Registration:**

Clinicaltrials.gov NCT03113955; registered April 14, 2017.

*Trial Registration* Clinicaltrials.gov NCT03113955; registered April 14, 2017.

**Level of evidence:**

2, Prospective, Non-randomized, Single-arm, study.

**Graphical Abstract:**

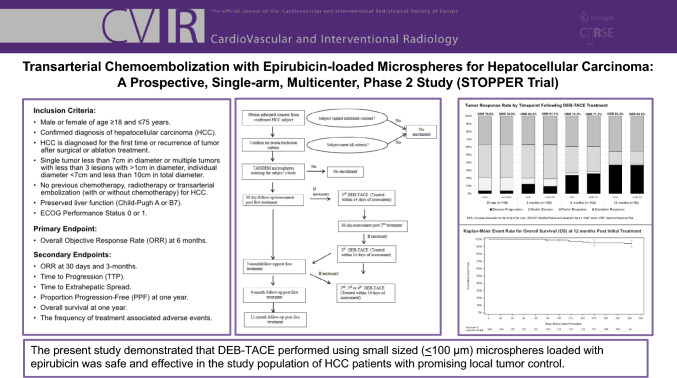

**Supplementary Information:**

The online version contains supplementary material available at 10.1007/s00270-024-03666-4.

## Introduction

Transarterial chemoembolization (TACE) has been recommended as the first treatment option for treating patients with hepatocellular carcinoma (HCC) diagnosed with barcelona clinic liver cancer (BCLC) Stage B [1, 2]. In addition, it has also been used to treat patients with BCLC Stage 0–A HCC who are not suitable candidates for, or unwilling to undergo, other treatments [3]. While conventional TACE (cTACE) has been widely investigated and survival benefit demonstrated [4–6], it can also be associated with adverse reactions such as post-embolization syndrome, liver damage, or ineffective, rapid release of cytotoxic agents [7, 8]. Drug-eluting beads transarterial chemoembolization (DEB-TACE) was developed as an alternative treatment for HCC patients with the advantage of being able to release drug in a sustained manner over time, thus reducing the risk of chemotherapy entering the systemic circulation [9]. Although higher objective response, better disease control, and fewer adverse events (AEs), with the exception of biliary tract injury, have been shown with DEB-TACE [10], a survival benefit compared to cTACE has not been demonstrated [11, 12].

Differing sizes of beads used for DEB-TACE have demonstrated variable levels of therapeutic efficacy and safety profiles [13]. Bead size, drug loading and drug release capabilities all have an impact on the outcomes associated with DEB-TACE. Treatment with medium-size (300–500 μm) microspheres has been reported to have similar efficacy and a better safety profile than with small-size (100–300 μm) microspheres [14]. A recent retrospective cohort study reported no significant difference in tumor response between 70–150 μm and 100–300 μm beads [15]. Smaller-sized microspheres have been shown to penetrate deeper into the fine tumor-feeding arteries, resulting in greater drug distribution and a higher density of microspheres within the tumor, resulting in a more predictable and homogenous tumor coverage [16, 17].

DEB-TACE with smaller-sized beads enables more distal and smaller size artery embolization and provides higher peak plasma concentration, area under the concentration–time curve, and tumor response rate when compared with large-sized beads. [16, 18]. TANDEM microspheres (Varian Medical Systems, Palo Alto, CA) are tightly calibrated and associated with an improved pharmacokinetic profile compared to other drug-eluting beads [17, 19, 20]. These beads have the capability of loading up to 50 mg of drug/mL microspheres and are available in three sizes (40, 75 and 100 μm) in diameter. They have demonstrated the ability to penetrate deep into HCC tumors in large quantities, resulting in satisfactory tumor necrosis following DEB-TACE using 40 μm doxorubicin-loaded beads [21, 22]. Two separate prospective and retrospective studies have also demonstrated the effectiveness and safety profile of DEB-TACE using doxorubicin-loaded TANDEM microspheres in HCC patients [23, 24]. Next to doxorubicin, epirubicin is the second most commonly used chemotherapeutic agent for DEB-TACE [18, 25, 26]. The use of epirubicin-loaded microspheres < 150 μm (DC BeadM1®, Boston Scientific, Marlborough, MA) showed promising results in the treatment of HCC without substantial hepatic toxicity [27].

To date, there has been limited data available reporting on clinical outcomes with DEB-TACE using epirubicin-loaded TANDEM microspheres, especially in patients with Hepatitis B (HBV)-related HCC. As a result, the STOPPER trial was prospectively conducted to assess the effectiveness and safety of using smaller sizes of these proprietary microspheres loaded with epirubicin for DEB-TACE in patients with HCC.

## Materials and Methods

This was a prospective, single-arm, multicenter trial conducted in patient with HCC at ten clinical centers in China with enrollment between October 24, 2017 and December 7, 2018. The study was approved by the Institutional Review Board/Ethics Committee of the participating study centers (Identifier: 2017ZDSYLL022-P01) and registered with clinicaltrials.gov (Identifier: NCT03113955). The study also conformed with Good Clinical Practice (CGP) guidelines, the Declaration of Helsinki, and applicable local laws. Each patient provided written informed consent before enrollment.

### Study Population

Baseline screening included patient demographics, physical exam, tumor and treatment history, Child–Pugh score, Eastern Cooperative Oncology Group (ECOG) evaluation, electrocardiogram, non-contrast and contrast-enhanced MRI to evaluate intrahepatic tumors, non-contrast chest CT scan to exclude extrahepatic lesions, and laboratory tests. Laboratory tests included routine blood tests, hepatic, renal and coagulation function tests, hepatitis testing, alpha-fetoprotein, and a pregnancy test, if appropriate.

Inclusion criteria for the study were patients aged 18–75 years diagnosed with HCC according to the diagnostic criteria for liver cancer in the Guidelines for Diagnosis and Treatment of Primary Liver Cancer in China in 2017 [28]. Additional inclusion criteria were preserved liver function (Child–Pugh class A or B7), an Eastern Cooperative Oncology Group (ECOG) performance status score of 0 or 1, a single tumor < 7 cm in the diameter or multiple tumors with maximum three lesions with individual diameters < 7 cm and a total diameter < 10 cm [29, 30], diagnosed with HCC for the first time or recurrence after curative-intent therapy (liver resection or ablation), and no history of chemotherapy, radiotherapy, or transarterial embolization for HCC.

Patients were excluded from the study if they had vascular invasion, extrahepatic spread, diffuse lesions (tumor burden of the whole liver > 50%), combined with arteriovenous shunt, invasion of the main trunk or primary branch of the portal vein. Patients were excluded if they had advanced liver disease (bilirubin levels > 2 mg/dL, aspartate aminotransferase, or alanine aminotransferase > 5 times the upper limit of normal value), renal failure, insufficient renal function (creatinine levels > 2 mg/dL) or any contraindications for TACE, epirubicin administration or magnetic resonance imaging (MRI) examination. In addition, pregnant women, breastfeeding women, or those who were unable to use reliable contraceptives during treatment or within 12 months after the end of the treatment and have participated in the trials of other drugs or devices within the past 30 days were excluded. Patients were also excluded based on the judgment of the investigators.

### DEB-TACE Procedure and Follow-up Assessments

Standard DEB-TACE procedures were performed by senior interventional radiologists in each center. DEB-TACE procedures were routinely performed with cone-beam CT guidance. The volume and size of epirubicin-loaded microspheres was based on the safety of the treatment, the tumor size and/or the vascular structure with the dose of epirubicin at the discretion of the treating physician. In patients with large tumor (> 5 cm), or with potential risk of arterioportal or hepatic venous shunting, 100 μm microspheres would be recommended. In patients with small tumor (< 3 cm) and without arterioportal or hepatic venous shunting, 40 μm microspheres would be recommended. Otherwise, 75 μm microspheres would be considered. Evaluation of procedure and device-related adverse events was performed peri-operatively, and follow-up assessments were performed 30 days, 3 months, 6 months and 12 months after the first DEB-TACE procedure.

Follow-up assessment included physical examination, ECOG and Child–Pugh scores, laboratory tests, electrocardiogram (at 3 months), non-contrast and contrast-enhanced MRI for evaluation of intrahepatic tumors, and non-contrast chest CT scan, concomitant medications prescribed since the last follow-up, adverse events that occurred since the previous assessment, and survival status (at 6 and 12 months).

Every 30 days following DEB-TACE treatment, each site conducted a post-treatment evaluation and determined whether another DEB-TACE procedure was necessary. If deemed necessary, the next treatment was completed within 14 days after the follow-up procedure with up to four DEB-TACE treatments permitted for each patient within 6 months following the first treatment. If the patient received four embolization treatments, the 30 days post-treatment evaluation was not performed and the 6 month follow-up after the first treatment would be conducted instead.

### Study Outcomes

The primary effectiveness outcome measure was 6-month objective response rate (ORR), which was defined as the percentage of patients who achieved either complete response (complete disappearance of lesions) or partial response. An independent Core Laboratory blindly assessed tumor response using EASL and mRECIST criteria (at baseline, 30-day, 3-month, 6-month and 12-month post-initial treatment) based on the plain scan and enhanced MRI performed. Thirty days after each DEB-TACE treatment, the site conducted a post-treatment evaluation and determined whether the next DEB-TACE treatment would be performed according to the patient’s condition.

The secondary outcome measures included 30-day ORR, 3-month ORR, time to tumor progression (TTP), time to extrahepatic spread, 12-month progression-free survival (PFS), 12-month overall survival rate, and the frequency of treatment-associated AEs or serious adverse events (SAEs), and the latter having a Grade 3 or higher score using based on the Common Terminology Criteria for Adverse Events (v5.0). ORR was assessed based on MRI using both the European Association for the Study of the Liver (EASL) and the modified Response Evaluation Criteria in Solid Tumors (mRECIST) criteria [31, 32]. TTP was defined as the length of time from the initiation of treatment to either the date of first disease progression or the date of death due to any cause, whichever occurred earlier. Time to extrahepatic spread was defined as the length of time from the initiation of treatment to the development of extrahepatic spread via imaging assessment.

### Statistical Analysis

The primary hypothesis was that the ORR for the DEB-TACE procedure at 6 months for the present study would exceed the performance goal (PG). The null (*H*_0_) and the alternative (*H*_1_) hypotheses for this primary outcome measure were as follows: *H*_0_: *P*_e_ ≤ PG; *H*_1_: *P*_e_ > PG; where *P*_e_ was the expected 6-month ORR (%). A standard approximation test was used to assess the one-sided hypotheses of the primary outcome measure, considering the superiority of the DEB-TACE procedure to PG, if the one-sided lower 97.5 confidence bound ORR at 6 months for the DEB-TACE procedure was more significant than PG. The sample size was determined based on the primary effectiveness outcome measure, considering a power of ≥ 85.0%, a one-sided significance level of *α* = 2.5%, a PG for 6 month ORR = 45.0%, and an expected 6 month ORR of 60.0%, resulting in a total of 98 patients being required for the 6 month assessment. Considering an assumed attrition rate of 10.0% (loss to the 6-month follow-up), it was determined that approximately 109 patients needed to be enrolled in the study.

The primary and prespecified additional outcome measures were analyzed on the intent-to-treat and the per-protocol basis, whereas the secondary outcome measures were analyzed on the per-protocol basis. To control the interobserver variability, data from independent core laboratory were used for the analysis of MRI results for tumor response. Statistical analyses were performed by the independent center (IQVIA, Durham, NC, USA). The descriptive variables statistics were presented as *n* (%), and the continuous variables were presented as median (interquartile range [IQR], range, or 95% confidence interval [CI]). The Kaplan–Meier analyses were performed on time to event indicators, including overall survival rate, TTP, and time to extrahepatic spread. All statistical analyses were performed using the Statistical Analysis Software (SAS), version 9.2 or later (Copyright© 2002–2010 by SAS Institute Inc., Cary, North Carolina 27513, USA).

## Results

### Demographics and Tumor Characteristics

**Fig. 1:**
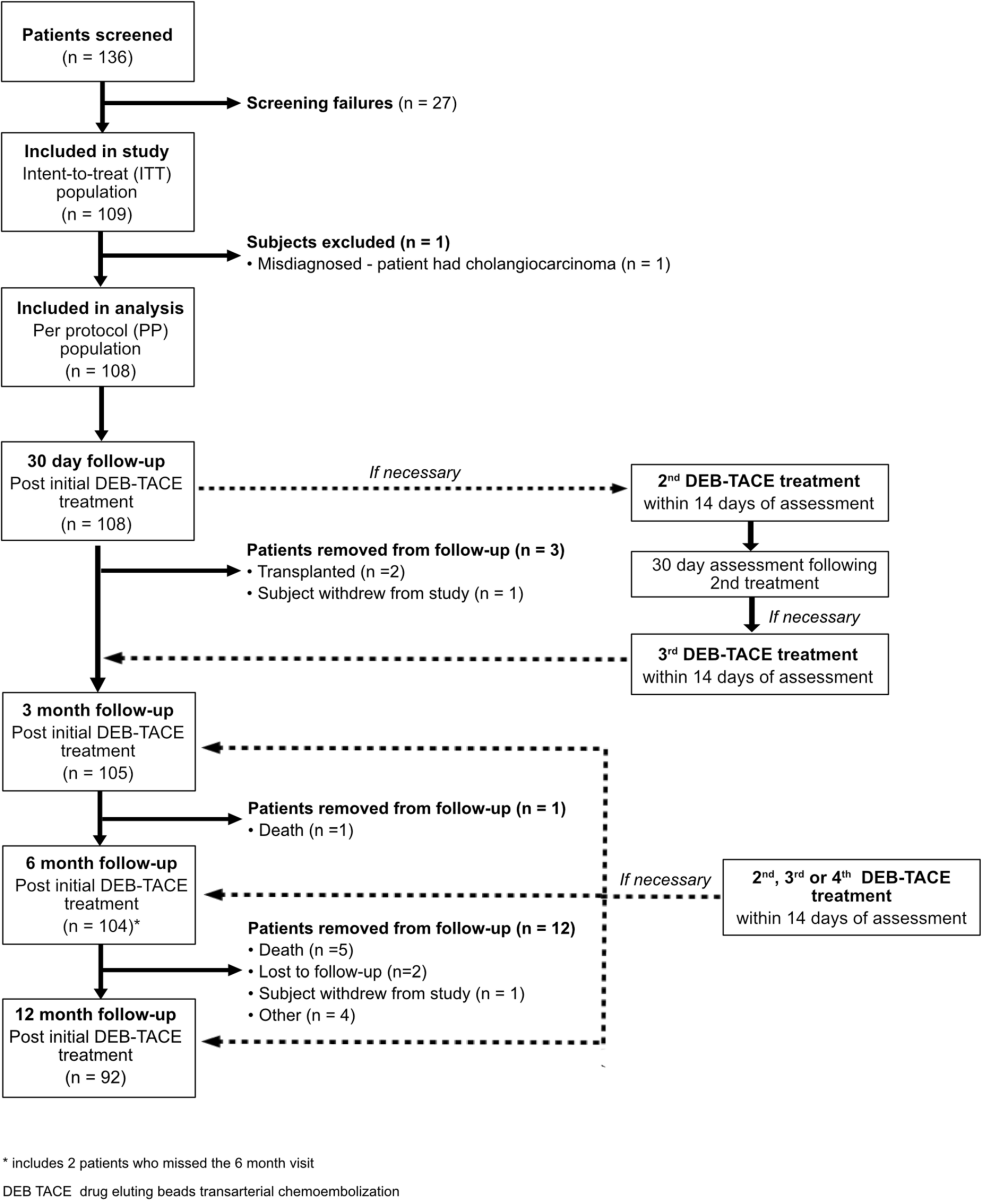
Clinical trial patient flow diagram

A total of 109 patients with HCC were enrolled and included in the intent-to-treat population (Fig. 1). One patient who was diagnosed with cholangiocarcinoma instead of HCC after enrollment and the first DEB-TACE procedure and not included in the final per-protocol analysis reported below. All 108 patients in the per-protocol group completed the 30 day follow-up assessment. Three (2.8%) of the 108 patients were not eligible for the 3 month follow-up due to either undergoing transplantation (*n* = 2) or withdrawal from the study (*n* = 1). At 6 months, an additional three patients were not available for follow-up as a result of death, failure to complete imaging evaluation, and lost to follow-up resulting in data for 102 (94.4%) patients being assessed at 6 months. Ninety-two patients completed the 12-month follow-up. Baseline characteristics for the per-protocol population are reported in Table 1. Patients had a mean of 1.56 tumors with 36 (33.3%) having multiple tumors.

**Table 1:** Demographics, medical history, disease characteristics, DEB-TACE treatments (*N* = 108)

Characteristic	Overall (*N* = 108)
Age (year; mean [range])	60.2 (31–75)
*Gender*	
Male	87 (80.6%)
Female	21 (19.4%)
*Medical history*	
Liver cirrhosis	44 (40.7%)
Hepatitis B infection	90 (83.3%)
History of Hepatitis C infection	8 (7.4%)
Diabetes mellitus	16 (14.8%)
History of smoking	45 (41.7%)
History of drinking	44 (40.7%)
Hyperlipidemia	1 (0.9%)
Hypertension	25 (23.1%)
Chronic obstructive pulmonary disease	2 (1.9%)
Coronary artery disease/coronary intervention/congestive heart failure	7 (6.5%)
Cerebrovascular accident	3 (2.8%)
Renal insufficiency/renal percutaneous intervention	2 (1.9%)
Peripheral vascular intervention	2 (1.9%)
*HCC diagnosis*	
Histological	18 (16.7%)
Clinical	90 (83.3%)
*CNLC stage*	
Ia	58 (53.7%)
Ib	31 (28.7%)
IIb	19 (17.6%)
*BCLC stage*	
0	10 (9.3%)
A	69 (63.9%)
B	18 (16.7%)
C*	11 (10.2%)
*ECOG performance status score*	
0	97 (89.8%)
1	11 (10.2%)
*Child–Pugh score*	
5	83 (76.9%)
6	14 (13.0%)
7	11 (10.2%)
*AFP (ng/mL)*	
< 200	84 (77.8%)
≥ 200	24 (22.2%)
*Total diameter of tumor(s)(cm)*	
< 3	34 (31.5%)
≥ 3 to < 5	41 (38.0%)
≥ 5 to < 7	22 (20.4%)
≥ 7 to < 10	11 (10.2%)
*Number of target lesions*	
1	70 (64.8%)
2	24 (22.2%)
3 or more	14 (12.9%)
*Previous treatment for HCC*	
Primary without previous treatment	94 (87.0%)
Recurrence after ablation (radiofrequency or microwave or laser)	8 (7.4%)
Recurrence after resection	9 (8.3%)

Data are presented as *n* (%) unless otherwise indicated

*AFP* alpha-fetoprotein, *BCLC* barcelona clinic liver cancer, *CNLC* china liver cancer, *DEB-TACE* drug-eluting beads transarterial chemoembolization, *ECOG* eastern cooperative oncology group, *HCC* hepatocellular carcinoma

*Patients in this stage presented with ECOG performance status of 1, no vascular invasion or extrahepatic spread and had preserved liver function at baseline

### Embolization with Epirubicin-Loaded Microspheres

The 108 patients in the per-protocol group underwent a total of 199 DEB-TACE procedures. Forty-two (38.9%) had a single DEB-TACE procedure and 47 (43.5%), 13 (12.0%) and six (5.6%) patients underwent two, three or four total DEB-TACE procedures respectively following the initial treatment.

The characteristics of embolization treatment in this study are shown in Table 2. For the first treatment, 24 (22.2%) of patients received 40 μm microspheres, 67 (62.1%) received 75 μm microspheres and 24 (22.3%) received 100 μm microspheres. Sixty-six patients received a second treatment. Except for two patients who received two different-sized (100 μm/2 ml and 100 μm/3 ml; 40 μm/3 ml and 75 μm/3 ml) microspheres during the same procedure, other patients just received the same-sized microspheres in both treatments. Hence, 15 (22.7%), 35 (53.1%) and 18 (27.2%) received 40 μm, 75 μm and 100 μm microspheres, respectively (Table 2). For the 19 patients undergoing a third treatment of 19 patients, 15.8%, 63.1%, and 21.1% received 40 μm, 75 μm and 100 μm microspheres. For the six patients having a fourth treatment 16.7%, 33.3% and 50.0% received 40 μm, 75 μm and 100 μm microspheres. The epirubicin dose was similar across all treatment groups ranging from 47.3 ± 14.0 mg to 52.5 ± 17.6 mg (Table 2). Bilateral hepatic arterial chemoembolization was performed in five (4.6%), five (7.6%), one (5.3%), and two (33.3%) of patients for procedures cycles 1, 2, 3, and 4, respectively. Unilateral hepatic artery chemoembolization was performed for the remainder of the patients in the study. There were no reports of device defects for any of the procedures.

**Table 2:** Characteristics of embolization treatment

Size / syringe volume	First treatment *N* = 108	Second treatment *N* = 66	Third treatment *N* = 19	Fourth treatment *N* = 6
*Bead size / syringe volume*				
40 μm / 2 mL	13.9% (15/108)	10.6% (7/66)	0.0% (0/19)	0.0% (0/6)
75 μm / 2 mL	27.8% (30/108)	36.4% (24/66)	36.8% (7/19)	33.3% (2/6)
100 μm / 2 mL	5.6% (6/108)	13.6% (9/66)	5.3% (1/19)	16.7% (1/6)
40 μm / 3 mL	8.3% (9/108)	12.1% (8/66)	15.8% (3/19)	16.7% (1/6)
75 μm / 3 mL	34.3% (37/108)	16.7% (11/66)	26.3% (5/19)	0.0% (0/6)
100 μm / 3 mL	16.7% (18/108)	13.6% (9/66)	15.8% (3/19)	33.3% (2/6)
Epirubicin dose, mg ± standard deviation (range)	52.5 ± 17.6 (15, 100)	47.3 ± 14.0 (20, 80)	51.1 ± 9.4 (30, 60)	51.7 ± 7.5 (40, 60)
*Treatment of lesions*				
Target lesion 1	100.0% (108/108)	95.5% (63/66)	84.2% (16/19)	100.0% (6/6)
Target lesion 2	30.6% (33/108)	18.2% (12/66)	21.1% (4/19)	16.7% (1/6)
Target lesion 3	9.3% (10/108)	3.0% (2/66)	5.3% (1/19)	0.0% (0/6)
Non-target lesion 1	0.0% (0/108)	4.5% (3/66)	10.5% (2/19)	0.0% (0/6)
Non-target lesion 2	0.0% (0/108)	1.5% (1/66)	5.3% (1/19)	0.0% (0/6)

Sixty-six patients received second treatments. Except two patients received two different-sized (100 μm/2 ml and 100 μm/3 ml; 40 μm/3 ml and 75 μm/3 ml) microspheres during the same procedure, other patients just received the same-sized microspheres in both treatments. Hence, 15 (22.7%), 35 (53.1%) and 18 (27.2%) of them received 40, 75 and 100 μm microspheres, respectively

### Efficacy and Safety Outcomes

**Fig. 2:**
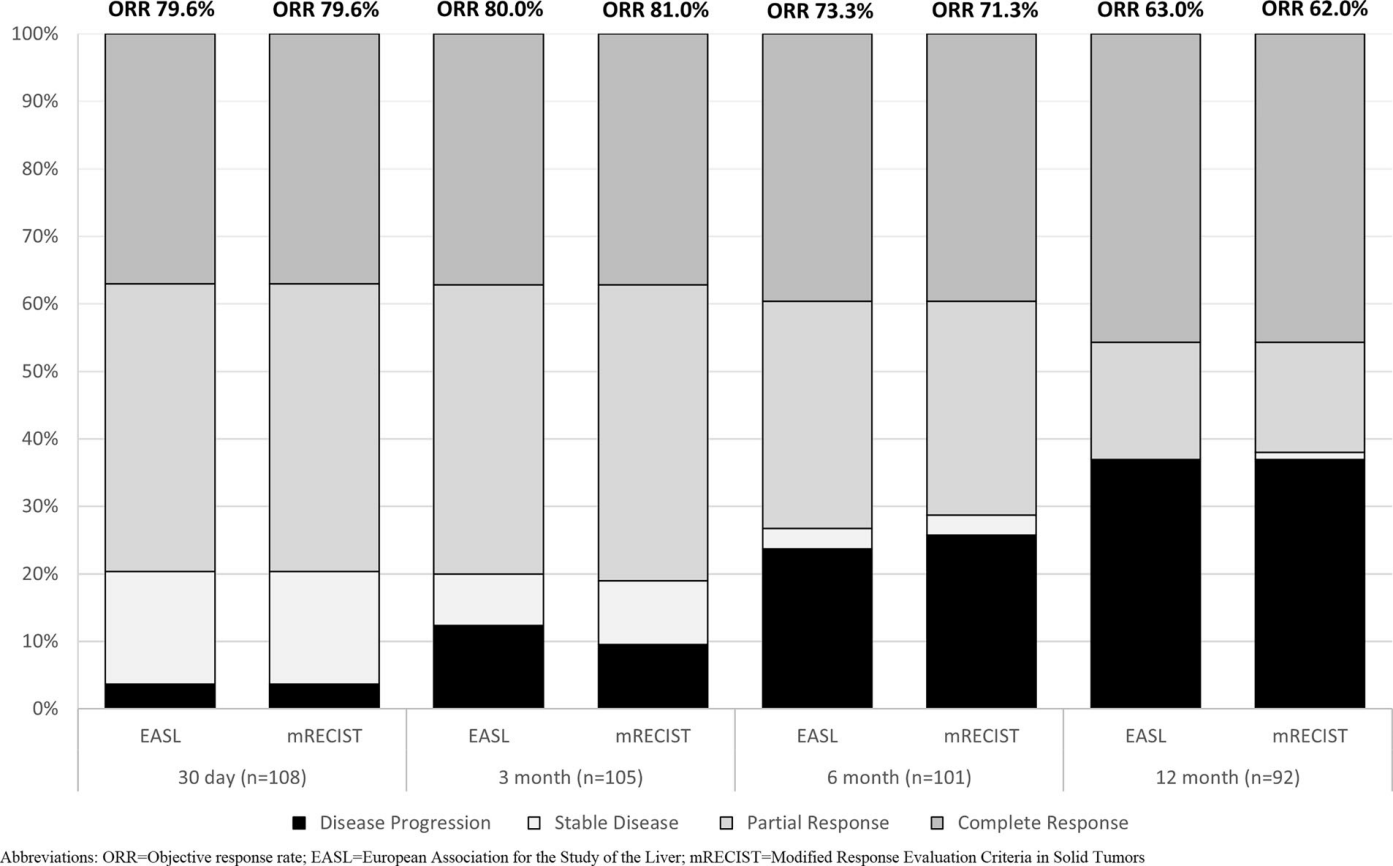
Tumor response rate by timepoint

**Fig. 3:**
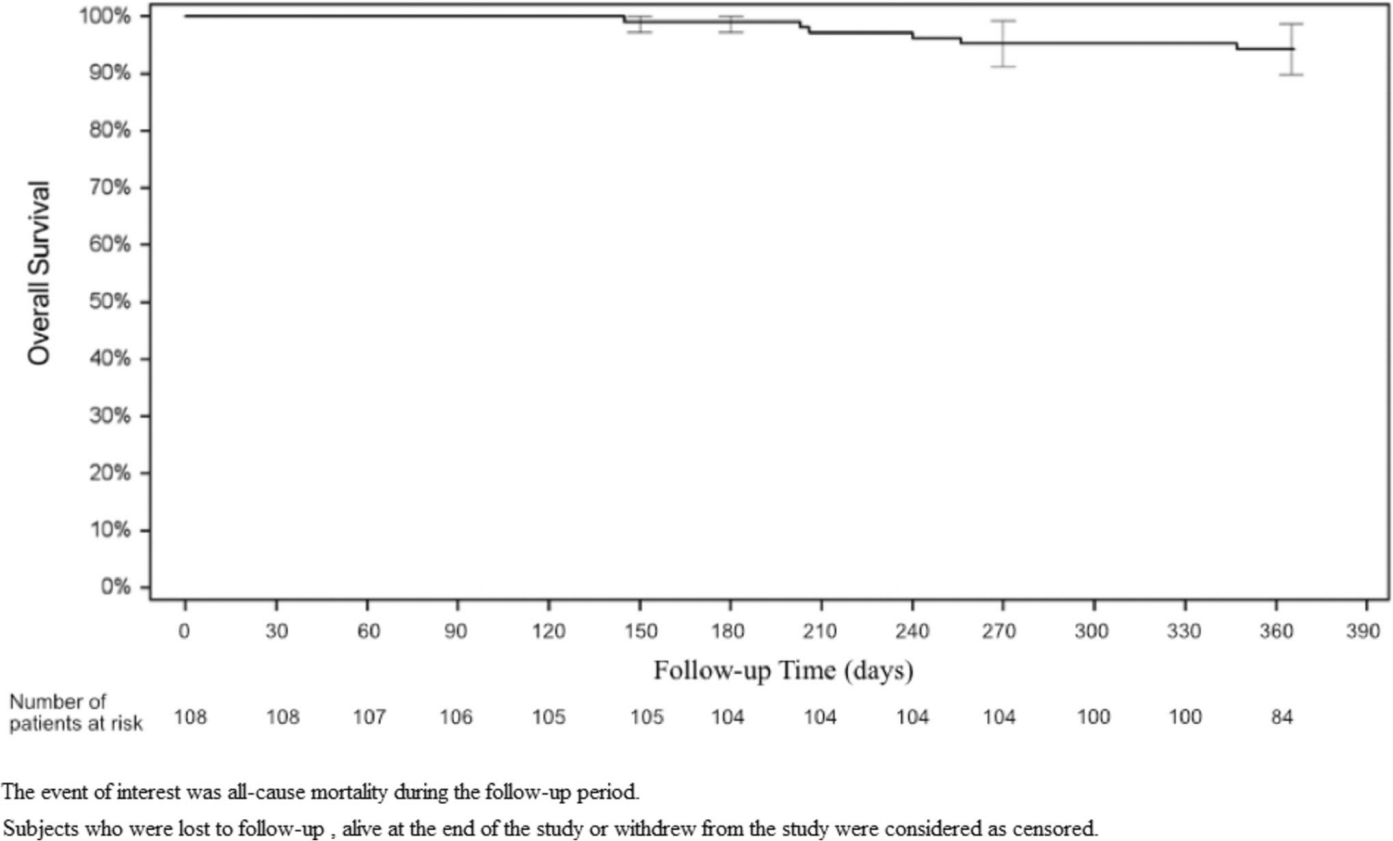
Kaplan–Meier event rate for overall survival (OS) at 12-month post-initial treatment

Six-month ORR was 73.3% based on the EASL criteria, and 71.3% based on mRECIST criteria (Fig. 2). The 30-day ORR was 79.6% for both criteria and the 3-month ORR was 80.0 or 81.0% for the EASL and mRECIST criteria, respectively. The complete response, partial response, stable disease, and progressive disease rates were also comparable for both criteria. Supplement Table 1 reports tumor measurements at baseline and by follow-up visit. The 12-month overall survival rate (Fig. 3) was 94.3% (95% CI 89.9–98.8%). Sixteen patients were censored prior to 12 months. This included seven deaths and nine patients who were withdrawn from the study, including two patients who received a transplant, two patients who were lost to follow-up, two patient who withdrew from the study and three patients for whom no 12-month follow-up data was reported.

The median TTP and median time to extrahepatic spread were all not reached (NR; 95% CI NR–NR). The 12-month probabilities for TTP were 60.7% (95% CI 51.9–71.1%) using the EASL criteria and 61.8% (95% CI 53.0–72.1%) using the mRECIST criteria. At the 6-month follow-up, the extrahepatic spread was observed in 9.5% of patients and increased to 21.1% at the 12-month follow-up. After tumor progression or extrahepatic spread, 48.1% (52/108) of patients received subsequent single or combined therapies (Table 4). Supplemental Tables 2 and 3 report Child–Pugh scores and ECOG performance status at baseline and by follow-up visit.

**Table 3:** Serious adverse events (SAEs)

Relationship	Not related		Unlikely related		Likely related		Very likely related		Definitely related	
	*N*	Incidence per patient	*N*	Incidence per patient	*N*	Incidence per patient	*N*	Incidence per patient	*N*	Incidence per patient
Total	109	68.5% (74/108)	20	5.6% (6/108)	15	2.8% (3/108)	9	1.9% (2/108)	6	1.9% (2/108)
Microspheres	36	22.2% (24/108)	8	5.6% (6/108)	4	2.8% (3/108)	3	1.9% (2/108)	2	1.9% (2/108)
Epirubicin	37	23.1% (25/108)	7	4.6% (5/108)	4	2.8% (3/108)	3	1.9% (2/108)	2	1.9% (2/108)
Procedure	36	22.2% (24/108)	5	4.6% (5/108)	7	4.6% (5/108)	3	1.9% (2/108)	2	1.9% (2/108)

There were no unanticipated adverse device events during the study. Six (5.5%) deaths were reported in the study population during the observation period. This included two deaths due to liver cancer metastases to the whole body and one each due to liver cancer, liver cancer metastasis to bone, cerebral hemorrhage, and biliary obstruction. No deaths were considered to be device or procedure related.

There was a total of 709 treatment-associated AEs reported during the 12-month post-initial procedure study observation period. The most commonly reported AEs include 132 reports of Grade 1 or 2 post-embolization syndrome, 58 complaints of abdominal pain, 26 incidences of pyrexia, 24 complaints of constipation, 14 instances of abnormal hepatic function, and 14 incidences of abdominal distension.

A total of 53 SAEs were reported within 12 months after the DEB-TACE procedure (Supplement Table 4), of which 30 were reported to be likely to definitely relate to the treatment (Table 3). This included 9 each reported for the microspheres and epirubicin and 12 procedure related SAEs. None of these resulted in death. SAEs reported as being likely to definitely related to the microspheres included five liver abscesses, and one each liver cancer recurrence, ascites, anemia, and post-embolization syndrome.

**Table 4:** Summary of subsequent cancer therapies

Therapy	Percent (number of patients)*
Any therapy	48.1% (52/108)
TACE	63.5% (33/52)
Radiofrequency ablation	13.5% (7/52)
Microwave ablation	11.5% (6/52)
Targeted biologic agent	9.6% (5/52)
Liver resection	5.8% (3/52)
Hepatic artery infused chemotherapy	3.8% (2/52)
Immunotherapy	3.8% (2/52)
External beam radiation	1.9% (1/52)
Stereotactic body radiation therapy	1.9% (1/52)
Other	25.0% (13/52)

*TACE* Transarterial chemotherapy embolization

*Total of individual therapies is greater than 100% since some patients had more than one subsequent treatment

## Discussion

The results of the present study demonstrate the safety and effectiveness of small-sized epirubicin-loaded microspheres when used for DEB-TACE in the treatment of HCC. The 73.3 and 71.3% 6-month ORR based on EASL and mRECIST criteria, respectively, exceeded the primary effectiveness performance goal of 45% which was reported in PRECISION V study [33].

These results exceeded the 6-month ORR of 45% resulting from the use of TACE in liver cancer patients reported for the PRECISION V international, multi-center randomized controlled trial which enrolled 212 patients at 19 centers in Europe [33]. The main selection criteria for patients enrolled in the present study were comparable to the PRECISION V study, including the BCLC stage, Child–Pugh score, and ECOG performance status. The PRECISION V trial confirmed that DEB-TACE can offer a greater rate of tumor response compared with cTACE with a better safety profile.

Several small retrospective and single-center studies have been conducted to explore the efficacy of DEB-TACE in differing HCC populations using the microspheres selected for the present study [34, 35]. The results of this trial are in line with previous studies. Greco et.al, reported an ORR of 72.6% (mRECIST criteria) with DEB-TACE using 40 µm microspheres preloaded with 100 mg of doxorubicin in HCC patients with early to intermediate stage disease [22]. Similarly, an ORR of 74.5% was observed in a prospective study of DEB-TACE using 100 mg doxorubicin-loaded microspheres after a median follow-up time of 21.2 months in patients with unresectable HCC [36]. The 12-month survival rate of 94.3% was similar to the 92.3% survival rate associated with the use of 150 or 100 mg doxorubicin-loaded microspheres [24], 95.8% survival rate using 75 mg doxorubicin-loaded 30–60 μm microspheres [36]*,* and 89.5% survival rate using 100 mg doxorubicin-loaded microspheres [37]. These results were better than the 56% one-year overall survival rate and 73% one-year survival in patients without ascites at baseline following the use of 75 μm microspheres loaded with 150 mg doxorubicin in locally untreatable HCC patients [38].

Similar to the present study, the median time to extrahepatic spread was not reached in any of the arms (sorafenib plus DEB-TACE versus placebo plus DEB-TACE) in the SPACE trial with a similar TTP in both the arms (169 versus 166 days) [39]. Despite this, the 60.7 and 61.8% 12-month probability for TTP using the EASL and mRECIST criteria, respectively, and the 78.9% 12 month extrahepatic spread probability provide insight on the efficacy of DEB-TACE using epirubicin-loaded microspheres for local tumor control.

With comparable treatment efficacy and safety, doxorubicin and epirubicin are the most commonly used agents in TACE for HCC [40, 41], DEB-TACE with epirubicin-loaded CalliSpheres® beads were demonstrated to be effective and well-tolerated in HCC patients [42]. Epirubicin loaded 100–500 μm microspheres has also been used for DEB-TACE [25, 26]. Epirubicin was used instead of doxorubicin as the chemotherapeutic agent for DEB-TACE for the present study since epirubicin is widely used in the clinic, with comparable safety in DEB-TACE for HCC [43].

There were no unanticipated AEs related to the procedure or therapy observed. Only 30 of the 709 (4.2%) were considered as SAEs likely to definitely associated with the procedure including 9 SAEs possibly, very likely or definitely related with the microspheres (Supplement Tables 5–7). The most common SAE likely to definitely associated with the microsphere was hepatic abscess which was reported for five patients. This demonstrates that DEB-TACE using epirubicin-loaded TANDEM microspheres in Chinese patients with HCC patients is safe, even with the high positive HBV rate (83.3%) noted in the study population. The safety results reported for the present study are similar to those from a study of patients with BCLC Stage A–B HCC treated 40 µm TANDEM microspheres preloaded with 100 mg doxorubicin did not experience any major complications [22].

Limitations for the study include the use of a single-arm trial design without a control group, the subjective choice of bead size and dose for DEB-TACE treatments, the lack of an analysis of the impact of bead size and dose across the differing types and size of tumors, and the limited 12-month follow-up period. There was also no analysis performed to correlate the impact of the number of DEB-TACE or other subsequent treatments on the outcome or the incidence and severity of AEs.

In conclusion, the present study demonstrated that DEB-TACE performed using small-sized (≤ 100 μm) TANDEM microspheres loaded with epirubicin was effective and safe in the study population of patients with promising local tumor control.

### Supplementary Information

Below is the link to the electronic supplementary material.

Supplementary file1 (DOCX 98 kb)

## Abbreviations

AEs: Adverse events
AFP: Alpha-fetoprotein
BCLC: Barcelona clinic liver cancer
CI: Confidence interval
CNLC: China liver cancer staging
cTACE: Conventional TACE
DEB-TACE: Drug-eluting beads transarterial chemoembolization
EASL: European association for the study of the liver
ECOG: Eastern cooperative oncology group
HCC: Hepatocellular carcinoma
IQR: Interquartile range
mRECIST: Modified response evaluation criteria in solid tumors
MRI: Magnetic resonance imaging
ORR: Objective response rate
OS: Overall survival
PFS: Progression-free survival
PG: Performance goal
SAEs: Serious adverse events
SAS: Statistical analysis software
