# Supplementary material for: Transarterial Chemoembolization with Epirubicin-Loaded Microspheres for Hepatocellular Carcinoma: A Prospective, Single-Arm, Multicenter, Phase 2 Study (STOPPER Trial)
Source: Cardiovasc Intervent Radiol. 2024 Feb 27;47(3):325–36. doi: 10.1007/s00270-024-03666-4 (PMC10920424; doi:10.1007/s00270-024-03666-4)
Supplement: Supplementary file 1 — Supplementary file1 (DOCX 98 kb) [file 270_2024_3666_MOESM1_ESM.docx]

**Supplement Table 1** Summary of lesion site and measurements by visit

|  | Baseline | 30 Day (N=108) | 3 Month (N=105) | 6 Month (N=102) | 12 Month (N=92) |
| --- | --- | --- | --- | --- | --- |
| *Target lesions (number)* | 162 | 162 | 157 | 151 | 135 |
| Target lesion 1 | 67.5% (108/160) | 67.5% (108/160) | 67.1% (104/155) | 68.5% (102/149) | 69.2% (92/133) |
| Target lesion 2 | 23.8% (38/160) | 23.8% (38/160) | 23.9% (37/155) | 22.8% (34/149) | 22.6% (30/133) |
| Target lesion 3 | 8.8% (14/160) | 8.8% (14/160) | 9.0% (14/155) | 8.7% (13/149) | 8.3% (11/133) |
| *Site of Lesion* |  |  |  |  |  |
| I - Caudate Section | 1.2% (2/162) | 1.2% (2/162) | 1.3% (2/157) | 1.3% (2/151) | 1.5% (2/135) |
| II - Left Lateral Section | 2.5% (4/162) | 2.5% (4/162) | 2.5% (4/157) | 2.6% (4/151) | 3.0% (4/135) |
| III - Left Lateral Section | 3.1% (5/162) | 3.1% (5/162) | 2.5% (4/157) | 2.6% (4/151) | 3.0% (4/135) |
| IV - Left Medial Section | 13.0% (21/162) | 14.2% (23/162) | 13.4% (21/157) | 14.6% (22/151) | 9.6% (13/135) |
| V - Right Anterior Section | 19.1% (31/162) | 19.1% (31/162) | 20.4% (32/157) | 19.9% (30/151) | 20.0% (27/135) |
| VI - Right Posterior Section | 18.5% (30/162) | 17.9% (29/162) | 17.2% (27/157) | 17.9% (27/151) | 17.8% (24/135) |
| VII - Right Posterior Section | 14.8% (24/162) | 15.4% (25/162) | 15.9% (25/157) | 15.9% (24/151) | 16.3% (22/135) |
| VIII - Right Anterior Section | 27.8% (45/162) | 26.5% (43/162) | 26.8% (42/157) | 25.2% (38/151) | 28.9% (39/135) |
| *Lesion Measurements* |  |  |  |  |  |
| Lesion Length, mm ± StD (range) | 28.4±15.3 (10.0, 67.0) | 9.5±13.0 (0.0, 69.0) | 10.0±13.8 (0.0, 76.0) | 11.1±21.3 (0.00, 158.00) | 11.7±20.8 (0.00, 125.00) |
| Lesion Width, mm ± StD (range) | 23.2±12.8 (5.0, 62.0) | 6.9±10.1 (0.0, 57.0) | 7.2±10.0 (0.0, 58.0) | 7.9±14.8 (0.0, 103.0) | 8.8±16.6 (0.0, 123.0) |
| Lesion Bidimensional measurement, mm^2^ ± StD (range) | 845.7±912.6 (65.0, 4030.0) | 192.3±454.9 (0.0, 3933.0) | 204.7±518.7 (0.0, 4408.0) | 395.12±691.5 (0.0, 16274.0) | 432.0±-1579.7 (0.0, 15375.0) |
| Sum of Longest Viable Tumor Diameter, mm ± StD (range) | 48.7±22.6 (11.0, 99.0) | 16.1±17.7 (0.0, 81.0) | 16.6±19.7 (0.0, 95.0) | 17.9±24.9 (0.0, 158.0) | 22.02±34.71 (0.0, 148.0) |
| Sum of Viable Tumor Bidimensional measurements, mm^2^ [number] (range) | 1327.8±1023.8  [162]  (65.0, 4164.0) | 475.5±642.1  [107] (15.0, 3933.0) | 522.0±706.2  [96] (30.0, 4408.0) | 877.8±2141.0  [90] (25.0, 16274.0) | 1418.1±2559.4  [73] (63.0, 15375.0) |
| *Non-Target Lesions* |  |  |  |  |  |
| Percent of subjects with non-target lesions (number) | 12.0% (13/108) | 12.0% (13/108) | 15.4% (16/104) | 20.4% (21/103) | 26.8% (26/97) |
| Number of non-target lesions | 16 | 16 | 21 | 36 | 60 |
| *Site of Lesion* |  |  |  |  |  |
| I - Caudate Section | 0.0% (0/13) | 0.0% (0/13) | 0.0% (0/16) | 0.0% (0/21) | 0.0% (0/26) |
| II - Left Lateral Section | 0.0% (0/13) | 0.0% (0/13) | 0.0% (0/16) | 0.0% (0/21) | 7.7% (2/26) |
| III - Left Lateral Section | 15.4% (2/13) | 15.4% (2/13) | 12.5% (2/16) | 19.0% (4/21) | 11.5% (3/26) |
| IV - Left Medial Section | 23.1% (3/13) | 23.1% (3/13) | 25.0% (4/16) | 23.8% (5/21) | 19.2% (5/26) |
| V - Right Anterior Section | 0.0% (0/13) | 0.0% (0/13) | 0.0% (0/16) | 4.8% (1/21) | 7.7% (2/26) |
| VI - Right Posterior Section | 15.4% (2/13) | 15.4% (2/13) | 12.5% (2/16) | 14.3% (3/21) | 23.1% (6/26) |
| VII - Right Posterior Section | 30.8% (4/13) | 30.8% (4/13) | 31.3% (5/16) | 19.0% (4/21) | 30.8% (8/26) |
| VIII - Right Anterior Section | 30.8% (4/13) | 30.8% (4/13) | 31.3% (5/16) | 33.3% (7/21) | 26.9% (7/26) |

StD Standard deviation

**Supplement Table 2** Summary of Child-Pugh Score by Visit

|  | Baseline (N=108) | 30 Day (N=108) | 3 Month (N=105) | 6 Month (N=102) | 12 Month (N=92) |
| --- | --- | --- | --- | --- | --- |
| *Serum Bilirubin* |  |  |  |  |  |
| <34.2umol/L (score = 1) | 98.1%  (106/108) | 94.3%  (100/106) | 91.3%  (95/104) | 89.0%  (89/100) | 87.0%  (80/92) |
| 34.2-51.3umol/L (score = 2) | 1.9%  (2/108) | 4.7%  (5/106) | 6.7%  (7/104) | 7.0%  (7/100) | 9.8%  (9/92) |
| >51.3umol/L (score = 3) | 0.0%  (0/108) | 0.9%  (1/106) | 1.9%  (2/104) | 4.0%  (4/100) | 3.3%  (3/92) |
| *Serum Albumin* |  |  |  |  |  |
| >35g/L (score = 1) | 86.1%  (93/108) | 95.3%  (101/106) | 89.4%  (93/104) | 90.0%  (90/100) | 95.7%  (88/92) |
| 28-35g/L (score = 2) | 13.9%  (15/108) | 3.8%  (4/106) | 10.6%  (11/104) | 9.0%  (9/100) | 3.3%  (3/92) |
| <28g/L (score = 3) | 0.0%  (0/108) | 0.9%  (1/106) | 0.0%  (0/104) | 1.0%  (1/100) | 1.1%  (1/92) |
| *INR* |  |  |  |  |  |
| <1.7 (score = 1) | 98.1% (106/108) | 99.1%  (105/106) | 99.0% (103/104) | 99.0%  (99/100) | 97.8%  (90/92) |
| 1.7-2.30 (score = 2) | 1.9%  (2/108) | 0.9%  (1/106) | 0.0%  (0/104) | 1.0%  (1/100) | 2.2%  (2/92) |
| >2.30 (score = 3) | 0.0%  (0/108) | 0.0%  (0/106) | 1.0%  (1/104) | 0.0%  (0/100) | 0.0%  (0/92) |
| *Ascites* |  |  |  |  |  |
| None (score = 1) | 84.3%  (91/108) | 87.7%  (93/106) | 82.7%  (86/104) | 79.0%  (79/100) | 73.9%  (68/92) |
| Mild (score = 2) | 15.7%  (17/108) | 12.3%  (13/106) | 17.3%  (18/104) | 20.0%  (20/100) | 23.9%  (22/92) |
| Severe (score = 3) | 0.0%  (0/108) | 0.0%  (0/106) | 0.0%  (0/104) | 1.0%  (1/100) | 2.2%  (2/92) |
| *Hepatic Encephalopathy* |  |  |  |  |  |
| None (score = 1) | 100.0% (108/108) | 100.0% (106/106) | 100.0% (104/104) | 100.0% (100/100) | 100.0%  (92/92) |
| Grade I-II (score = 2) | 0.0%  (0/108) | 0.0%  (0/106) | 0.0%  (0/104) | 0.0%  (0/100) | 0.0%  (0/92) |
| Grade III-IV (score = 3) | 0.0%  (0/108) | 0.0%  (0/106) | 0.0%  (0/104) | 0.0%  (0/100) | 0.0%  (0/92) |
| *Score* |  |  |  |  |  |
| 5 | 76.9% (83/108) | 80.2%  (85/106) | 69.2%  (72/104) | 70.0%  (70/100) | 67.4%  (62/92) |
| 6 | 13.0% (14/108) | 16.0%  (17/106) | 21.2%  (22/104) | 17.0%  (17/100) | 22.8%  (21/92) |
| 7 | 10.2% (11/108) | 1.9%  (2/106) | 9.6%  (10/104) | 8.0%  (8/100) | 5.4%  (5/92) |
| 8 | 0.0%  (0/108) | 1.9%  (2/106) | 0.0%  (0/104) | 4.0%  (4/100) | 1.1%  (1/92) |
| 9 | 0.0%  (0/108) | 0.0%  (0/106) | 0.0%  (0/104) | 1.0%  (1/100) | 2.2%  (2/92) |
| 10 | 0.0%  (0/108) | 0.0%  (0/106) | 0.0%  (0/104) | 0.0%  (0/100) | 0.0%  (0/92) |
| 11 | 0.0%  (0/108) | 0.0%  (0/106) | 0.0%  (0/104) | 0.0%  (0/100) | 1.1%  (1/92) |
| *Class* |  |  |  |  |  |
| A | 89.8% (97/108) | 96.2%  (102/106) | 90.4%  (94/104) | 87.0%  (87/100) | 90.2%  (83/92) |
| B | 10.2% (11/108) | 3.8%  (4/106) | 9.6%  (10/104) | 13.0%  (13/100) | 8.7%  (8/92) |
| C | 0.0%  (0/108) | 0.0%  (0/106) | 0.0%  (0/104) | 0.0%  (0/100) | 1.1%  (1/92) |
| INR International normalized ratio  Class A: 5-6 scores, B: 7-9 scores, C: 10-15 scores. | | | | | |

**Supplemental Table 3** Summary of ECOG Performance Status by Visit

| ECOG Performance Status | Baseline (N=108) | 30 Day (N=108) | 3 Month (N=105) | 6 Month (N=102) | 12 Month (N=92) |
| --- | --- | --- | --- | --- | --- |
| 0 | 89.8% (97/108) | 84.1% (90/107) | 82.9% (87/105) | 80.4% (82/102) | 88.0% (81/92) |
| 1 | 10.2% (11/108) | 15.9% (17/107) | 16.2% (17/105) | 17.6% (18/102) | 12.0% (11/92) |
| 2 | 0.0% (0/108) | 0.0% (0/107) | 0.0% (0/105) | 0.0% (0/102) | 0.0% (0/92) |
| 3 | 0.0% (0/108) | 0.0% (0/107) | 1.0% (1/105) | 0.0% (0/102) | 0.0% (0/92) |
| 4 | 0.0% (0/108) | 0.0% (0/107) | 0.0% (0/105) | 2.0% (2/102) | 0.0% (0/92) |
| 5 | 0.0% (0/108) | 0.0% (0/107) | 0.0% (0/105) | 0.0% (0/102) | 0.0% (0/92) |

**Supplemental Table 4** Listing of Serious Adverse Event

| **Subject ID** | **MedDRA Preferred Term** | **Days*** | **Action taken** | **Outcome** |
| --- | --- | --- | --- | --- |
| 0873S005 | Hypersplenism | 149 | Medication given or regimen adjusted; Surgery | Recovering/ Resolving |
| 0873S005 | Gastrointestinal haemorrhage | 152 | Surgery; Medical procedure | Recovered/ Resolved |
| 0879S003 | Gastric varices | 161 | Medication given or regimen adjusted; In-patient hospitalization/prolonged hospitalization; Surgery | Recovered/ Resolved |
| 0879S003 | Varices oesophageal | 363 | In-patient hospitalization/prolonged hospitalization | Recovered/ Resolved |
| 0879S004 | Liver abscess | 7 | In-patient hospitalization/prolonged hospitalization; Other | Recovered/ Resolved |
| 0879S005 | Gastrointestinal haemorrhage | 120 | In-patient hospitalization/prolonged hospitalization | Recovered/ Resolved |
| 0879S005 | Oesophageal varices haemorrhage | 137 | In-patient hospitalization/prolonged hospitalization | Recovered/ Resolved |
| 2134S002 | Haemangioma of liver | 237 | In-patient hospitalization/prolonged hospitalization; Other | Recovered/ Resolved |
| 2309S003 | Fracture | 339 | In-patient hospitalization/prolonged hospitalization; Other | Recovering/ Resolving |
| 2309S008 | Atrioventricular block complete | 257 | In-patient hospitalization/prolonged hospitalization; Surgery | Recovered/ Resolved |
| 2309S009 | Cardiac failure | 3 | In-patient hospitalization/prolonged hospitalization | Recovered/ Resolved |
| 2309S012 | Liver abscess | 60 | In-patient hospitalization/prolonged hospitalization | Recovered/ Resolved |
| 2309S012 | Cholecystitis acute | 138 | Medication given or regimen adjusted; In-patient hospitalization/prolonged hospitalization | Recovered/ Resolved |
| 2309S012 | Hepatocellular carcinoma | 240 | None | Fatal |
| 2309S013 | Hypersplenism | 0 | Surgery | Recovering/ Resolving |
| 2309S013 | Hepatocellular carcinoma | 144 | Medication given or regimen adjusted; In-patient hospitalization/prolonged hospitalization; Surgery | Recovered/ Resolved |
| 2309S016 | Goitre | 126 | In-patient hospitalization/prolonged hospitalization; Surgery | Recovered/ Resolved |
| 3730S004 | Disease progression | 405 | None | Fatal |
| 3730S005 | Headache | 13 | Medication given or regimen adjusted; In-patient hospitalization/prolonged hospitalization | Recovered/ Resolved |
| 3730S006 | Metastatic neoplasm | 56 | Medication given or regimen adjusted; In-patient hospitalization/prolonged hospitalization; Surgery | Recovered/ Resolved |
| 3730S006 | Hepatic cancer metastatic | 147 | Medication given or regimen adjusted; In-patient hospitalization/prolonged hospitalization | Fatal |
| 3732S005 | Bile duct obstruction | 145 | None | Fatal |
| 3732S010 | Liver abscess | 100 | In-patient hospitalization/prolonged hospitalization; Other | Recovered/ Resolved |
| 3732S010 | Liver abscess | 177 | In-patient hospitalization/prolonged hospitalization | Recovered/ Resolved |
| 3732S010 | Liver abscess | 292 | In-patient hospitalization/prolonged hospitalization | Recovered/ Resolved |
| 3732S015 | Cerebral haemorrhage | 176 | In-patient hospitalization/prolonged hospitalization | Fatal |
| 3732S021 | Liver abscess | 105 | Medication given or regimen adjusted; In-patient hospitalization/prolonged hospitalization | Recovered/ Resolved |
| 3733S006 | Oesophageal squamous cell carcinoma | 35 | Medication given or regimen adjusted; In-patient hospitalization/prolonged hospitalization | Not recovered/ Not resolved |
| 3914S007 | Duodenal ulcer haemorrhage | 316 | In-patient hospitalization/prolonged hospitalization | Unknown |

*Days were calculated based on AE Onset date – Initial Treatment date.

MedDRA, Medical Dictionary for Regulatory Activities

**Supplement Table 5** Frequency of Site-reported Serious Adverse Events - Relationship to Microspheres (n=108)

|  | | Not Related | | Unlikely Related | | Possible Relationship | | Probable Relationship | | Causal Relationship | |
| --- | --- | --- | --- | --- | --- | --- | --- | --- | --- | --- | --- |
| Serious Adverse Event | | Events | Rate of Subjects with Event | Events | Rate of Subjects with Event | Events | Rate of Subjects with Event | Events | Rate of Subjects with Event | Events | Rate of Subjects with Event |
| Total | | 36 | 22.2% (24/108) | 8 | 5.6% (6/108) | 4 | 2.8% (3/108) | 3 | 1.9% (2/108) | 2 | 1.9% (2/108) |
| Hepatic cancer metastatic | | 2 | 1.9% (2/108) | 0 | 0.0% (0/108) | 0 | 0.0% (0/108) | 0 | 0.0% (0/108) | 0 | 0.0% (0/108) |
| Hepatocellular carcinoma | | 2 | 1.9% (2/108) | 0 | 0.0% (0/108) | 0 | 0.0% (0/108) | 0 | 0.0% (0/108) | 0 | 0.0% (0/108) |
| Metastases to bone | | 2 | 0.9% (1/108) | 0 | 0.0% (0/108) | 0 | 0.0% (0/108) | 0 | 0.0% (0/108) | 0 | 0.0% (0/108) |
| Hemangioma of liver | | 1 | 0.9% (1/108) | 0 | 0.0% (0/108) | 0 | 0.0% (0/108) | 0 | 0.0% (0/108) | 0 | 0.0% (0/108) |
| Hepatic cancer | | 1 | 0.9% (1/108) | 0 | 0.0% (0/108) | 0 | 0.0% (0/108) | 0 | 0.0% (0/108) | 0 | 0.0% (0/108) |
| Hepatic cancer recurrent | | 0 | 0.0% (0/108) | 0 | 0.0% (0/108) | 1 | 0.9% (1/108) | 0 | 0.0% (0/108) | 0 | 0.0% (0/108) |
| Metastatic neoplasm | | 0 | 0.0% (0/108) | 1 | 0.9% (1/108) | 0 | 0.0% (0/108) | 0 | 0.0% (0/108) | 0 | 0.0% (0/108) |
| Esophageal squamous cell carcinoma | | 1 | 0.9% (1/108) | 0 | 0.0% (0/108) | 0 | 0.0% (0/108) | 0 | 0.0% (0/108) | 0 | 0.0% (0/108) |
| Gastrointestinal hemorrhage | | 2 | 1.9% (2/108) | 1 | 0.9% (1/108) | 0 | 0.0% (0/108) | 0 | 0.0% (0/108) | 0 | 0.0% (0/108) |
| Ascites | | 0 | 0.0% (0/108) | 0 | 0.0% (0/108) | 1 | 0.9% (1/108) | 0 | 0.0% (0/108) | 0 | 0.0% (0/108) |
| Duodenal ulcer hemorrhage | | 0 | 0.0% (0/108) | 1 | 0.9% (1/108) | 0 | 0.0% (0/108) | 0 | 0.0% (0/108) | 0 | 0.0% (0/108) |
| Gastric varices | | 1 | 0.9% (1/108) | 0 | 0.0% (0/108) | 0 | 0.0% (0/108) | 0 | 0.0% (0/108) | 0 | 0.0% (0/108) |
| Intestinal obstruction | | 1 | 0.9% (1/108) | 0 | 0.0% (0/108) | 0 | 0.0% (0/108) | 0 | 0.0% (0/108) | 0 | 0.0% (0/108) |
| Esophageal varices hemorrhage | | 1 | 0.9% (1/108) | 0 | 0.0% (0/108) | 0 | 0.0% (0/108) | 0 | 0.0% (0/108) | 0 | 0.0% (0/108) |
| Upper gastrointestinal hemorrhage | | 1 | 0.9% (1/108) | 0 | 0.0% (0/108) | 0 | 0.0% (0/108) | 0 | 0.0% (0/108) | 0 | 0.0% (0/108) |
| Varices esophageal | 1 | 0.9% (1/108) | 0 | 0.0% (0/108) | 0 | 0.0% (0/108) | 0 | 0.0% (0/108) | 0 | 0.0% (0/108) |  |
| Liver abscess | 1 | 0.9% (1/108) | 1 | 0.9% (1/108) | 1 | 0.9% (1/108) | 2 | 0.9% (1/108) | 2 | 1.9% (2/108) |  |
| Lung infection | 1 | 0.9% (1/108) | 0 | 0.0% (0/108) | 0 | 0.0% (0/108) | 0 | 0.0% (0/108) | 0 | 0.0% (0/108) |  |
| Pneumonia | 1 | 0.9% (1/108) | 0 | 0.0% (0/108) | 0 | 0.0% (0/108) | 0 | 0.0% (0/108) | 0 | 0.0% (0/108) |  |
| Sepsis | 0 | 0.0% (0/108) | 1 | 0.9% (1/108) | 0 | 0.0% (0/108) | 0 | 0.0% (0/108) | 0 | 0.0% (0/108) |  |
| Hypersplenism | 3 | 2.8% (3/108) | 0 | 0.0% (0/108) | 0 | 0.0% (0/108) | 0 | 0.0% (0/108) | 0 | 0.0% (0/108) |  |
| Anemia | 0 | 0.0% (0/108) | 0 | 0.0% (0/108) | 1 | 0.9% (1/108) | 0 | 0.0% (0/108) | 0 | 0.0% (0/108) |  |
| Splenomegaly | 1 | 0.9% (1/108) | 0 | 0.0% (0/108) | 0 | 0.0% (0/108) | 0 | 0.0% (0/108) | 0 | 0.0% (0/108) |  |
| Bile duct obstruction | 1 | 0.9% (1/108) | 0 | 0.0% (0/108) | 0 | 0.0% (0/108) | 0 | 0.0% (0/108) | 0 | 0.0% (0/108) |  |
| Cholecystitis acute | 1 | 0.9% (1/108) | 0 | 0.0% (0/108) | 0 | 0.0% (0/108) | 0 | 0.0% (0/108) | 0 | 0.0% (0/108) |  |
| Jaundice hepatocellular | 0 | 0.0% (0/108) | 1 | 0.9% (1/108) | 0 | 0.0% (0/108) | 0 | 0.0% (0/108) | 0 | 0.0% (0/108) |  |
| Liver disorder | 1 | 0.9% (1/108) | 0 | 0.0% (0/108) | 0 | 0.0% (0/108) | 0 | 0.0% (0/108) | 0 | 0.0% (0/108) |  |
| Atrioventricular block complete | 1 | 0.9% (1/108) | 0 | 0.0% (0/108) | 0 | 0.0% (0/108) | 0 | 0.0% (0/108) | 0 | 0.0% (0/108) |  |
| Cardiac failure | 1 | 0.9% (1/108) | 0 | 0.0% (0/108) | 0 | 0.0% (0/108) | 0 | 0.0% (0/108) | 0 | 0.0% (0/108) |  |
| Myocardial ischemia | 1 | 0.9% (1/108) | 0 | 0.0% (0/108) | 0 | 0.0% (0/108) | 0 | 0.0% (0/108) | 0 | 0.0% (0/108) |  |
| Cerebral hemorrhage | 1 | 0.9% (1/108) | 0 | 0.0% (0/108) | 0 | 0.0% (0/108) | 0 | 0.0% (0/108) | 0 | 0.0% (0/108) |  |
| Cerebral infarction | 1 | 0.9% (1/108) | 0 | 0.0% (0/108) | 0 | 0.0% (0/108) | 0 | 0.0% (0/108) | 0 | 0.0% (0/108) |  |
| Headache | 0 | 0.0% (0/108) | 1 | 0.9% (1/108) | 0 | 0.0% (0/108) | 0 | 0.0% (0/108) | 0 | 0.0% (0/108) |  |
| Disease progression | 1 | 0.9% (1/108) | 0 | 0.0% (0/108) | 0 | 0.0% (0/108) | 0 | 0.0% (0/108) | 0 | 0.0% (0/108) |  |
| Pyrexia | 0 | 0.0% (0/108) | 1 | 0.9% (1/108) | 0 | 0.0% (0/108) | 0 | 0.0% (0/108) | 0 | 0.0% (0/108) |  |
| Fracture | 1 | 0.9% (1/108) | 0 | 0.0% (0/108) | 0 | 0.0% (0/108) | 0 | 0.0% (0/108) | 0 | 0.0% (0/108) |  |
| Post embolization syndrome | 0 | 0.0% (0/108) | 0 | 0.0% (0/108) | 0 | 0.0% (0/108) | 1 | 0.9% (1/108) | 0 | 0.0% (0/108) |  |
| Vertigo | 1 | 0.9% (1/108) | 0 | 0.0% (0/108) | 0 | 0.0% (0/108) | 0 | 0.0% (0/108) | 0 | 0.0% (0/108) |  |
| Goiter | 1 | 0.9% (1/108) | 0 | 0.0% (0/108) | 0 | 0.0% (0/108) | 0 | 0.0% (0/108) | 0 | 0.0% (0/108) |  |
| Pleural effusion | 1 | 0.9% (1/108) | 0 | 0.0% (0/108) | 0 | 0.0% (0/108) | 0 | 0.0% (0/108) | 0 | 0.0% (0/108) |  |

“Events” numbers are total episodes of each type of event among all subjects.
"Rate of Subjects with Event" numbers are percent of subjects who experienced one or more episodes of the event.
“Events” numbers for “TOTAL” are the sum of the individual event category totals.
"Rate of Subjects with Event" numbers for “TOTAL” is the percent of subjects who experienced an adverse event.

**Supplement Table 6** Frequency of Site-reported Serious Adverse Events - Relationship to Epirubicin (n=108)

|  | | Not Related | | Unlikely Related | | Possible Relationship | | Probable Relationship | | Causal Relationship | | |
| --- | --- | --- | --- | --- | --- | --- | --- | --- | --- | --- | --- | --- |
| Serious Adverse Event | | Events | Rate of Subjects with Event | Events | Rate of Subjects with Event | Events | Rate of Subjects with Event | Events | Rate of Subjects with Event | Events | Rate of Subjects with Event | |
| Total | | 37 | 23.1% (25/108) | 7 | 4.6% (5/108) | 4 | 2.8% (3/108) | 3 | 1.9% (2/108) | 2 | 1.9% (2/108) | |
| Hepatic cancer metastatic | | 2 | 1.9% (2/108) | 0 | 0.0% (0/108) | 0 | 0.0% (0/108) | 0 | 0.0% (0/108) | 0 | 0.0% (0/108) | |
| Hepatocellular carcinoma | | 2 | 1.9% (2/108) | 0 | 0.0% (0/108) | 0 | 0.0% (0/108) | 0 | 0.0% (0/108) | 0 | 0.0% (0/108) | |
| Metastases to bone | | 2 | 0.9% (1/108) | 0 | 0.0% (0/108) | 0 | 0.0% (0/108) | 0 | 0.0% (0/108) | 0 | 0.0% (0/108) | |
| Hemangioma of liver | | 1 | 0.9% (1/108) | 0 | 0.0% (0/108) | 0 | 0.0% (0/108) | 0 | 0.0% (0/108) | 0 | 0.0% (0/108) | |
| Hepatic cancer | | 1 | 0.9% (1/108) | 0 | 0.0% (0/108) | 0 | 0.0% (0/108) | 0 | 0.0% (0/108) | 0 | 0.0% (0/108) | |
| Hepatic cancer recurrent | | 0 | 0.0% (0/108) | 0 | 0.0% (0/108) | 1 | 0.9% (1/108) | 0 | 0.0% (0/108) | 0 | 0.0% (0/108) | |
| Metastatic neoplasm | | 0 | 0.0% (0/108) | 1 | 0.9% (1/108) | 0 | 0.0% (0/108) | 0 | 0.0% (0/108) | 0 | 0.0% (0/108) | |
| Esophageal squamous cell carcinoma | | 1 | 0.9% (1/108) | 0 | 0.0% (0/108) | 0 | 0.0% (0/108) | 0 | 0.0% (0/108) | 0 | 0.0% (0/108) | |
| Gastrointestinal hemorrhage | | 2 | 1.9% (2/108) | 1 | 0.9% (1/108) | 0 | 0.0% (0/108) | 0 | 0.0% (0/108) | 0 | 0.0% (0/108) | |
| Ascites | | 0 | 0.0% (0/108) | 0 | 0.0% (0/108) | 1 | 0.9% (1/108) | 0 | 0.0% (0/108) | 0 | 0.0% (0/108) | |
| Duodenal ulcer hemorrhage | | 1 | 0.9% (1/108) | 0 | 0.0% (0/108) | 0 | 0.0% (0/108) | 0 | 0.0% (0/108) | 0 | 0.0% (0/108) | |
| Gastric varices | | 1 | 0.9% (1/108) | 0 | 0.0% (0/108) | 0 | 0.0% (0/108) | 0 | 0.0% (0/108) | 0 | 0.0% (0/108) | |
| Intestinal obstruction | | 1 | 0.9% (1/108) | 0 | 0.0% (0/108) | 0 | 0.0% (0/108) | 0 | 0.0% (0/108) | 0 | 0.0% (0/108) | |
| Esophageal varices hemorrhage | | 1 | 0.9% (1/108) | 0 | 0.0% (0/108) | 0 | 0.0% (0/108) | 0 | 0.0% (0/108) | 0 | 0.0% (0/108) | |
| Upper gastrointestinal hemorrhage | | 1 | 0.9% (1/108) | 0 | 0.0% (0/108) | 0 | 0.0% (0/108) | 0 | 0.0% (0/108) | 0 | 0.0% (0/108) | |
| Varices esophageal | 1 | 0.9% (1/108) | 0 | 0.0% (0/108) | 0 | 0.0% (0/108) | 0 | 0.0% (0/108) | 0 | 0.0% (0/108) | |  |
| Liver abscess | 1 | 0.9% (1/108) | 1 | 0.9% (1/108) | 1 | 0.9% (1/108) | 2 | 0.9% (1/108) | 2 | 1.9% (2/108) | |  |
| Lung infection | 1 | 0.9% (1/108) | 0 | 0.0% (0/108) | 0 | 0.0% (0/108) | 0 | 0.0% (0/108) | 0 | 0.0% (0/108) | |  |
| Pneumonia | 1 | 0.9% (1/108) | 0 | 0.0% (0/108) | 0 | 0.0% (0/108) | 0 | 0.0% (0/108) | 0 | 0.0% (0/108) | |  |
| Sepsis | 0 | 0.0% (0/108) | 1 | 0.9% (1/108) | 0 | 0.0% (0/108) | 0 | 0.0% (0/108) | 0 | 0.0% (0/108) | |  |
| Hypersplenism | 3 | 2.8% (3/108) | 0 | 0.0% (0/108) | 0 | 0.0% (0/108) | 0 | 0.0% (0/108) | 0 | 0.0% (0/108) | |  |
| Anemia | 0 | 0.0% (0/108) | 0 | 0.0% (0/108) | 1 | 0.9% (1/108) | 0 | 0.0% (0/108) | 0 | 0.0% (0/108) | |  |
| Splenomegaly | 1 | 0.9% (1/108) | 0 | 0.0% (0/108) | 0 | 0.0% (0/108) | 0 | 0.0% (0/108) | 0 | 0.0% (0/108) | |  |
| Bile duct obstruction | 1 | 0.9% (1/108) | 0 | 0.0% (0/108) | 0 | 0.0% (0/108) | 0 | 0.0% (0/108) | 0 | 0.0% (0/108) | |  |
| Cholecystitis acute | 1 | 0.9% (1/108) | 0 | 0.0% (0/108) | 0 | 0.0% (0/108) | 0 | 0.0% (0/108) | 0 | 0.0% (0/108) | |  |
| Jaundice hepatocellular | 0 | 0.0% (0/108) | 1 | 0.9% (1/108) | 0 | 0.0% (0/108) | 0 | 0.0% (0/108) | 0 | 0.0% (0/108) | |  |
| Liver disorder | 1 | 0.9% (1/108) | 0 | 0.0% (0/108) | 0 | 0.0% (0/108) | 0 | 0.0% (0/108) | 0 | 0.0% (0/108) | |  |
| Atrioventricular block complete | 1 | 0.9% (1/108) | 0 | 0.0% (0/108) | 0 | 0.0% (0/108) | 0 | 0.0% (0/108) | 0 | 0.0% (0/108) | |  |
| Cardiac failure | 1 | 0.9% (1/108) | 0 | 0.0% (0/108) | 0 | 0.0% (0/108) | 0 | 0.0% (0/108) | 0 | 0.0% (0/108) | |  |
| Myocardial ischemia | 1 | 0.9% (1/108) | 0 | 0.0% (0/108) | 0 | 0.0% (0/108) | 0 | 0.0% (0/108) | 0 | 0.0% (0/108) | |  |
| Cerebral hemorrhage | 1 | 0.9% (1/108) | 0 | 0.0% (0/108) | 0 | 0.0% (0/108) | 0 | 0.0% (0/108) | 0 | 0.0% (0/108) | |  |
| Cerebral infarction | 1 | 0.9% (1/108) | 0 | 0.0% (0/108) | 0 | 0.0% (0/108) | 0 | 0.0% (0/108) | 0 | 0.0% (0/108) | |  |
| Headache | 0 | 0.0% (0/108) | 1 | 0.9% (1/108) | 0 | 0.0% (0/108) | 0 | 0.0% (0/108) | 0 | 0.0% (0/108) | |  |
| Disease progression | 1 | 0.9% (1/108) | 0 | 0.0% (0/108) | 0 | 0.0% (0/108) | 0 | 0.0% (0/108) | 0 | 0.0% (0/108) | |  |
| Pyrexia | 0 | 0.0% (0/108) | 1 | 0.9% (1/108) | 0 | 0.0% (0/108) | 0 | 0.0% (0/108) | 0 | 0.0% (0/108) | |  |
| Fracture | 1 | 0.9% (1/108) | 0 | 0.0% (0/108) | 0 | 0.0% (0/108) | 0 | 0.0% (0/108) | 0 | 0.0% (0/108) | |  |
| Post embolization syndrome | 0 | 0.0% (0/108) | 0 | 0.0% (0/108) | 0 | 0.0% (0/108) | 1 | 0.9% (1/108) | 0 | 0.0% (0/108) | |  |
| Vertigo | 1 | 0.9% (1/108) | 0 | 0.0% (0/108) | 0 | 0.0% (0/108) | 0 | 0.0% (0/108) | 0 | 0.0% (0/108) | |  |
| Goiter | 1 | 0.9% (1/108) | 0 | 0.0% (0/108) | 0 | 0.0% (0/108) | 0 | 0.0% (0/108) | 0 | 0.0% (0/108) | |  |
| Pleural effusion | 1 | 0.9% (1/108) | 0 | 0.0% (0/108) | 0 | 0.0% (0/108) | 0 | 0.0% (0/108) | 0 | 0.0% (0/108) | |  |
| “Events” numbers are total episodes of each type of event among all subjects. "Rate of Subjects with Event" numbers are percent of subjects who experienced one or more episodes of the event. “Events” numbers for “TOTAL” are the sum of the individual event category totals. "Rate of Subjects with Event" numbers for “TOTAL” is the percent of subjects who experienced an adverse event. | | | | | | | | | | |  |  |

**Supplement Table 7** Frequency of Site-reported Serious Adverse Events - Relationship to Procedure (n=108)

|  | | Not Related | | Unlikely Related | | Possible Relationship | | Probable Relationship | | Causal Relationship | |
| --- | --- | --- | --- | --- | --- | --- | --- | --- | --- | --- | --- |
| Serious Adverse Event | | Events | Rate of Subjects with Event | Events | Rate of Subjects with Event | Events | Rate of Subjects with Event | Events | Rate of Subjects with Event | Events | Rate of Subjects with Event |
| Total | | 36 | 23.1% (25/108) | 5 | 4.6% (5/108) | 7 | 4.6% (5/108) | 3 | 1.9% (2/108) | 2 | 1.9% (2/108) |
| Hepatic cancer metastatic | | 2 | 1.9% (2/108) | 0 | 0.0% (0/108) | 0 | 0.0% (0/108) | 0 | 0.0% (0/108) | 0 | 0.0% (0/108) |
| Hepatocellular carcinoma | | 2 | 1.9% (2/108) | 0 | 0.0% (0/108) | 0 | 0.0% (0/108) | 0 | 0.0% (0/108) | 0 | 0.0% (0/108) |
| Metastases to bone | | 2 | 0.9% (1/108) | 0 | 0.0% (0/108) | 0 | 0.0% (0/108) | 0 | 0.0% (0/108) | 0 | 0.0% (0/108) |
| Hemangioma of liver | | 1 | 0.9% (1/108) | 0 | 0.0% (0/108) | 0 | 0.0% (0/108) | 0 | 0.0% (0/108) | 0 | 0.0% (0/108) |
| Hepatic cancer | | 1 | 0.9% (1/108) | 0 | 0.0% (0/108) | 0 | 0.0% (0/108) | 0 | 0.0% (0/108) | 0 | 0.0% (0/108) |
| Hepatic cancer recurrent | | 0 | 0.0% (0/108) | 0 | 0.0% (0/108) | 1 | 0.9% (1/108) | 0 | 0.0% (0/108) | 0 | 0.0% (0/108) |
| Metastatic neoplasm | | 0 | 0.0% (0/108) | 1 | 0.9% (1/108) | 0 | 0.0% (0/108) | 0 | 0.0% (0/108) | 0 | 0.0% (0/108) |
| Esophageal squamous cell carcinoma | | 1 | 0.9% (1/108) | 0 | 0.0% (0/108) | 0 | 0.0% (0/108) | 0 | 0.0% (0/108) | 0 | 0.0% (0/108) |
| Gastrointestinal hemorrhage | | 2 | 1.9% (2/108) | 1 | 0.9% (1/108) | 0 | 0.0% (0/108) | 0 | 0.0% (0/108) | 0 | 0.0% (0/108) |
| Ascites | | 0 | 0.0% (0/108) | 0 | 0.0% (0/108) | 1 | 0.9% (1/108) | 0 | 0.0% (0/108) | 0 | 0.0% (0/108) |
| Duodenal ulcer hemorrhage | | 1 | 0.9% (1/108) | 0 | 0.0% (0/108) | 0 | 0.0% (0/108) | 0 | 0.0% (0/108) | 0 | 0.0% (0/108) |
| Gastric varices | | 1 | 0.9% (1/108) | 0 | 0.0% (0/108) | 0 | 0.0% (0/108) | 0 | 0.0% (0/108) | 0 | 0.0% (0/108) |
| Intestinal obstruction | | 1 | 0.9% (1/108) | 0 | 0.0% (0/108) | 0 | 0.0% (0/108) | 0 | 0.0% (0/108) | 0 | 0.0% (0/108) |
| Esophageal varices hemorrhage | | 1 | 0.9% (1/108) | 0 | 0.0% (0/108) | 0 | 0.0% (0/108) | 0 | 0.0% (0/108) | 0 | 0.0% (0/108) |
| Upper gastrointestinal hemorrhage | | 1 | 0.9% (1/108) | 0 | 0.0% (0/108) | 0 | 0.0% (0/108) | 0 | 0.0% (0/108) | 0 | 0.0% (0/108) |
| Varices esophageal | 1 | 0.9% (1/108) | 0 | 0.0% (0/108) | 0 | 0.0% (0/108) | 0 | 0.0% (0/108) | 0 | 0.0% (0/108) |  |
| Liver abscess | 0 | 0.0% (0/108) | 0 | 0.0% (0/108) | 3 | 2.8% (3/108) | 2 | 0.9% (1/108) | 2 | 1.9% (2/108) |  |
| Lung infection | 1 | 0.9% (1/108) | 0 | 0.0% (0/108) | 0 | 0.0% (0/108) | 0 | 0.0% (0/108) | 0 | 0.0% (0/108) |  |
| Pneumonia | 1 | 0.9% (1/108) | 0 | 0.0% (0/108) | 0 | 0.0% (0/108) | 0 | 0.0% (0/108) | 0 | 0.0% (0/108) |  |
| Sepsis | 0 | 0.0% (0/108) | 0 | 0.0% (0/108) | 1 | 0.9% (1/108) | 0 | 0.0% (0/108) | 0 | 0.0% (0/108) |  |
| Hypersplenism | 3 | 2.8% (3/108) | 0 | 0.0% (0/108) | 0 | 0.0% (0/108) | 0 | 0.0% (0/108) | 0 | 0.0% (0/108) |  |
| Anemia | 0 | 0.0% (0/108) | 0 | 0.0% (0/108) | 1 | 0.9% (1/108) | 0 | 0.0% (0/108) | 0 | 0.0% (0/108) |  |
| Splenomegaly | 1 | 0.9% (1/108) | 0 | 0.0% (0/108) | 0 | 0.0% (0/108) | 0 | 0.0% (0/108) | 0 | 0.0% (0/108) |  |
| Bile duct obstruction | 1 | 0.9% (1/108) | 0 | 0.0% (0/108) | 0 | 0.0% (0/108) | 0 | 0.0% (0/108) | 0 | 0.0% (0/108) |  |
| Cholecystitis acute | 1 | 0.9% (1/108) | 0 | 0.0% (0/108) | 0 | 0.0% (0/108) | 0 | 0.0% (0/108) | 0 | 0.0% (0/108) |  |
| Jaundice hepatocellular | 0 | 0.0% (0/108) | 1 | 0.9% (1/108) | 0 | 0.0% (0/108) | 0 | 0.0% (0/108) | 0 | 0.0% (0/108) |  |
| Liver disorder | 1 | 0.9% (1/108) | 0 | 0.0% (0/108) | 0 | 0.0% (0/108) | 0 | 0.0% (0/108) | 0 | 0.0% (0/108) |  |
| Atrioventricular block complete | 1 | 0.9% (1/108) | 0 | 0.0% (0/108) | 0 | 0.0% (0/108) | 0 | 0.0% (0/108) | 0 | 0.0% (0/108) |  |
| Cardiac failure | 1 | 0.9% (1/108) | 0 | 0.0% (0/108) | 0 | 0.0% (0/108) | 0 | 0.0% (0/108) | 0 | 0.0% (0/108) |  |
| Myocardial ischemia | 1 | 0.9% (1/108) | 0 | 0.0% (0/108) | 0 | 0.0% (0/108) | 0 | 0.0% (0/108) | 0 | 0.0% (0/108) |  |
| Cerebral hemorrhage | 1 | 0.9% (1/108) | 0 | 0.0% (0/108) | 0 | 0.0% (0/108) | 0 | 0.0% (0/108) | 0 | 0.0% (0/108) |  |
| Cerebral infarction | 1 | 0.9% (1/108) | 0 | 0.0% (0/108) | 0 | 0.0% (0/108) | 0 | 0.0% (0/108) | 0 | 0.0% (0/108) |  |
| Headache | 0 | 0.0% (0/108) | 1 | 0.9% (1/108) | 0 | 0.0% (0/108) | 0 | 0.0% (0/108) | 0 | 0.0% (0/108) |  |
| Disease progression | 1 | 0.9% (1/108) | 0 | 0.0% (0/108) | 0 | 0.0% (0/108) | 0 | 0.0% (0/108) | 0 | 0.0% (0/108) |  |
| Pyrexia | 0 | 0.0% (0/108) | 1 | 0.9% (1/108) | 0 | 0.0% (0/108) | 0 | 0.0% (0/108) | 0 | 0.0% (0/108) |  |
| Fracture | 1 | 0.9% (1/108) | 0 | 0.0% (0/108) | 0 | 0.0% (0/108) | 0 | 0.0% (0/108) | 0 | 0.0% (0/108) |  |
| Post embolization syndrome | 0 | 0.0% (0/108) | 0 | 0.0% (0/108) | 0 | 0.0% (0/108) | 1 | 0.9% (1/108) | 0 | 0.0% (0/108) |  |
| Vertigo | 1 | 0.9% (1/108) | 0 | 0.0% (0/108) | 0 | 0.0% (0/108) | 0 | 0.0% (0/108) | 0 | 0.0% (0/108) |  |
| Goiter | 1 | 0.9% (1/108) | 0 | 0.0% (0/108) | 0 | 0.0% (0/108) | 0 | 0.0% (0/108) | 0 | 0.0% (0/108) |  |
| Pleural effusion | 1 | 0.9% (1/108) | 0 | 0.0% (0/108) | 0 | 0.0% (0/108) | 0 | 0.0% (0/108) | 0 | 0.0% (0/108) |  |

“Events” numbers are total episodes of each type of event among all subjects.
"Rate of Subjects with Event" numbers are percent of subjects who experienced one or more episodes of the event.
“Events” numbers for “TOTAL” are the sum of the individual event category totals.
"Rate of Subjects with Event" numbers for “TOTAL” is the percent of subjects who experienced an adverse event.
